# A ROCK1 Inhibitior Fasudil Alleviates Cardiomyocyte Apoptosis in Diabetic Cardiomyopathy by Inhibiting Mitochondrial Fission in a Type 2 Diabetes Mouse Model

**DOI:** 10.3389/fphar.2022.892643

**Published:** 2022-07-05

**Authors:** Xinhui Fan, Xiaoxing Li, Huiruo Liu, Feng Xu, Xiaoping Ji, Yuguo Chen, Chuanbao Li

**Affiliations:** ^1^ Department of Emergency Medicine and Chest Pain Center, Qilu Hospital of Shandong University, Cheeloo College of Medicine, Shandong University, Jinan, China; ^2^ Key Laboratory of Emergency and Critical Care Medicine of Shandong Province, Qilu Hospital, Shandong University, Jinan, China; ^3^ The Key Laboratory of Cardiovascular Remodeling and Function Research, Chinese Ministry of Education and Chinese Ministry of Public Health, Qilu Hospital, Shandong University, Jinan, China; ^4^ Department of Geriatrics, Qilu Hospital, Shandong University, Jinan, China

**Keywords:** type 2 diabetes, diabetic cardiomyopathy, fasudil, ROCK1, DRP1, mitochondrial fission

## Abstract

Diabetes mellitus (DM) often involves cardiovascular complications; however, treatment regimens are limited. ROCK1 (rho-associated coiled-coil containing protein kinase 1) serves as a pathological factor in several diabetic complications. Herein, we aimed to explore the effect of Fasudil (a ROCK1 inhibitor) on the progress of cardiac dysfunction in type 2 DM (T2DM), and to explore the possible mechanisms. Type II diabetic mice models were established by inducing insulin resistance through a high-fat diet combined with low-dose streptozotocin (STZ) injection. NMCMs (neonatal mouse ventricular cardiac myocytes) in the control group were treated with 5.5 mM glucose, while those in the High Glucose (HG) group were treated with 33 mM glucose and 10 nmol/L insulin. *In vivo*, we found that type 2 diabetes enhanced the expression and activation of ROCK1 (*p* < 0.05). The ROCK1 inhibitor, Fasudil, prevented cardiac dysfunction, fibrosis, oxidative stress and myocardial ultrastructural disorders (*p* < 0.05) in the diabetic mice. *In vitro*, ROCK1 was upregulated in HG-induced cardiomyocytes, and ROCK1 inhibition using Fasudil reversed the increased apoptosis, consistent with *in vivo* results. Mechanistically, ROCK1 inhibition abrogated apoptosis, relieved mitochondrial fission, and efficiently attenuated the escalated production of reactive oxygen species *in vitro* and *in vivo*. The content of Ser616-phosphorylated dynamin-related protein 1 (Drp1) increased while ROCK1 led to apoptosis in HG-treated cardiomyocytes, which could be partly neutralized by ROCK1 inhibition with Fasudil, consistent with the *in vivo* results. Fasudil attenuated the cardiac dysfunction in diabetes by decreasing excessive mitochondrial fission via inhibiting Drp1 phosphorylation at serine 616.

## Introduction

Diabetes mellitus (DM), especially type 2 DM (T2DM), is one of the most prevalent metabolic diseases globally. The DM epidemic has increased rapidly in recent years, and is currently estimated to affect 10.9% of the population in China (Wang et al., 2017) and 9.5% in the United States (Menke et al., 2015). Diabetic cardiomyopathy (DCM), the major cause of death in individuals with diabetes, is characterized by diastolic and systolic dysfunction in the absence of hypertension, coronary artery disease (CAD), or valvular heart disease (Falcão-Pires and Leite-Moreira, 2012; Shah and Brownlee, 2016). Peripheral insulin resistance, pancreatic β-cell loss and β-cell mass reduction, are the major causes of T2DM, which leads to uncontrolled glucose metabolism and chronic complications of multiple organs (Nyenwe et al., 2011). Apoptosis of cardiomyocytes, a pivotal pathological alteration of DCM (Kuethe et al., 2007), causes progressive decline in effective cardiac function (Foo et al., 2005). Hyperglycemia is a hallmark of DM metabolic disorders and plays an indispensable role in cardiomyocyte apoptosis (Falcão-Pires and Leite-Moreira, 2012).

RhoA regulates the structure of the actin cytoskeleton. RhoA and its subsequent target, ROCK (RhoA-associated kinase) are critical in mediating cell shape, adhesion, migration, proliferation, reactive oxygen species (ROS), and apoptosis (Chang et al., 2006; Shi et al., 2010; Majid et al., 2012). ROCK1 and ROCK2, the two isoforms of the ROCK family, have unique functions in cardiac remodeling and hypertrophy (Rikitake et al., 2005; Zhang et al., 2006; Shi et al., 2008). Fasudil is the only specific ROCK inhibitor currently available for the treatment of vasospasm after subarachnoid hemorrhage (Asano et al., 1987). In hypertrophic cardiomyopathy, *ROCK1* genetic deletion inhibited a variety of pathological incidents, including inhibition of cardiomyocyte apoptosis (Shi et al., 2010). However, the precise mechanisms by which ROCK1 affects DCM caused by type II diabetes is still unclear.

Mitochondria are important organelles in the production of energy. They undergo extensive fragmentation during the progression of cell apoptosis (Ni et al., 2016), and excessive mitochondrial fission can contribute to mitochondrial dysfunction, because it is linked to a partial drop in increased ROS production, mitochondrial membrane potential (MMP), and cellular apoptosis (Yu et al., 2006; Wada and Nakatsuka, 2016). Interestingly, an *in vitro* study demonstrated that mitochondrial fission, along with fusion processes, are essential for intracellular ROS generation and cell apoptosis under hyperglycemic conditions (Chiong et al., 2014). However, whether Fasudil could protect against DCM via decreasing mitochondrial fission remain unknown.

In the present study, we explored the mechanisms of Fasudil on cardiomyocyte apoptosis in type II diabetes-induced DCM. We also aimed to determine whether Fasudil alleviates myocardial fibrosis, oxidative stress and mitochondrial dysfunction. Ultimately, we determined that Fasudil prevents imbalanced mitochondrial fission via dynamin-related protein 1 (Drp1) phosphorylation at Ser616.

## Materials and Methods

### Cell Culture

Neonatal mouse ventricular cardiomyocytes (NMCMs) were isolated from the left ventricles of 3–7 day old C57BL/6N mice as documented previously (Liu et al., 2013). Cells were cultured in DMEM medium, augmented with 10% FBS, as well as 1% penicillin-streptomycin, and grown at 37°C under 5% CO_2_ conditions. To identify the impact of high glucose and high insulin, NMCMs were cultured in 5.5 mM glucose (control) or 33 mM glucose +10 nmol/L insulin (HG). ROCK1 inhibition was induced using Fasudil (100 μmol/L).

### HFD/STZ-Induced Type 2 Diabetic Mice

C57BL/6N male mice (6–8 weeks old) were purchased from Beijing Charles River (Beijing, China). After 1 week of acclimatization, we randomized the mice into four groups (n = 10 per group): Sham + saline, sham + Fasudil, DM + saline, and DM + Fasudil. The sham group received a normal diet, and the DM group was fed with a high-fat (HF) diet (45% fat and 1% cholesterol). After 8 weeks of feeding, diabetic C57/BL6n mice with insulin resistance were treated intraperitoneally with 50 mg/kg of streptozotocin (STZ) (S0130, Sigma-Aldrich) dispersed in 50 mM Trisodium citrate solution (pH 4.5) as documented previously (Poucher et al., 2012). The control mice were administered with citrate buffer. Type II diabetes was considered to have been developed when the mouse’s blood glucose was between 11.1 and 28.0 mM, and were used for the following experiments. Mice were intraperitoneal injected with saline or Fasudil (10 mg/kg, dissolved in saline) once daily, which was continued for 12 weeks. The HF diet was maintained during the STZ and the Fasudil treatment. All experimental procedures were conducted according to animal protocols approved by the Shandong University Animal Care and Use Committee.

### Intraperitoneal Glucose Tolerance Test (IPGTT) and Intraperitoneal Insulin Tolerance Test (IPITT)

After the mice were fasted for 12 h, glucose tolerance was evaluated by IPGTT. Mice were injected glucose (1 g/kg) intraperitoneally, and blood glucose were tested at 0, 15, 30, 60, and 120 min. Fasting blood glucose (FBG) was measured using a one-touch glucometer (Roche Diagnostics GmbH, Mannheim, Germany). IPITT was performed after 6 h of fasting to assess insulin tolerance. A bolus of insulin (1 unit/kg) was given intraperitoneally, and plasma glucose was measured as described above.

### Cell Proliferation Assay

Cell proliferation was measured by a Cell Counting Kit-8 (CCK8) assay (AR1160, Boster, Wuhan, China), in which absorbance at 450 nm is directly proportional to the number of living cells.

### Apoptosis Assay Analysis *in vitro* and *in vivo*

*In vitro*, flow cytometry was performed according to the manufacturer’s instructions (556,547, BD Biosciences). The fluorescences of propidium iodide (PI) and fluorescein isothiocyanate (FITC) were analyzed using the software FlowJo (FlowJo LLC). Cardiomyocyte apoptosis *in vivo* was assessed using an *in situ* apoptosis detection kit (S7110, Millipore, Billerica, MA, United States) by the indirect terminal deoxynulceotidyl transferase nick-end-labeling (TUNEL) method, according to the manufacturer’s instructions.

### Western Blotting

Proteins were separated through 12%–15% SDS-PAGE and transferred to polyvinylidene fluoride (PVDF) membranes. The membranes were incubated overnight at 4°C with antibodies against ROCK1 (1:1000, ab134181, Abcam), cleaved caspase-3 (1:1000, 9661, Cell Signaling Technology), Phospho-Myosin phosphatase targeting Thr853 (p-MYPT1) (1:1000, 4563, Cell Signaling Technology), 4-Hydroxynonenal (4-HNE)-protein adducts (1:3000, ab46545, Abcam), Cytochrome c (1:1000, 24,840, Cell Signaling Technology), cytochrome C oxidase subunit 4I1 (COX IV) (1:1000, 4850, Cell Signaling Technology), and β-actin (1:5000, 2D4H5, Proteintech). After washing, membranes were incubated with secondary antibodies. Bands were detected using chemiluminescence reagent. The intensity of the bands was quantified using ImageJ software.

### Echocardiography Measurements

Echocardiography was performed using the Vevo770TM echocardiography imaging system (VisualSonics, Toronto, Canada). Left ventricular ejection fraction (LVEF%), fractional shortening (FS%), peak E to peak A ratio (E/A), early (E′) to late (A′) diastolic velocity ratio (E’/A’), left ventricle end-diastolic posterior wall diameter (LVPWd), and left ventricle end-diastolic volume (LVVd) were measured using computer algorithms.

### Immunofluorescence Staining

Frozen heart sections were permeabilized in 0.1% Triton X-100 and blocked with goat serum, and then stained with anti-sarcomeric actin (SAB4200689, Sigma). Dylight 594-labeled anti-mouse secondary antibody (ab150116, Abcam) was incubated at 37°C in the dark for 1 h. Nuclei were labeled with DAPI. The images were acquired with a fluorescence microscope.

### Measurement of Myocardial Fibrosis

The left ventricular tissues were fixed in 4% neutral formaldehyde and embedded in paraffin to produce 5-μm sections. The sections were stained with Masson’s trichrome and Sirius red to detect collagen. The fibrotic area fraction was obtained by using automated image analysis (Image-Pro Plus, Media Cybernetics, Rockville, MD, United States).

### Transmission Electron Microscopy (TEM)

The LV tissue sections (70 nm) were viewed using a TEM (JEOL Ltd., Tokyo, Japan). Flameng grading was used to quantify the severity of mitochondrial structural damage, as described previously (Flameng et al., 1980), which uses specific criteria to assign a score from 0 to four to each mitochondrion, with higher scores indicating more injury.

### Determination of Cellular ROS and Cardiac ROS Production

The ROS levels in NMCMs were measured by incubation with 2ʹ,7ʹ-dichlorofluorescin diacetate (ab113851, DCFH-DA) (Abcam). Dihydroethidium (DHE) (S0063, Beyotime, Jiangsu, China) staining was used to detect intracellular superoxide anion levels in heart tissues. DAPI (ab104139, Abcam) was used for counterstaining.

### Glutathione (GSH), Malondialdehyde (MDA), and Superoxide Dismutase (SOD) Assays

Measurements of tissue GSH and MDA levels, and plasma SOD levels were performed using commercial assay kits (Jiancheng, A006-2, Nanjing, China; Jiancheng, A003-1, Nanjing, China; Jiancheng, A001-3, Nanjing, China, respectively). All experiments were performed according to the manufacturer’s instructions.

### Mitochondrial Membrane Potential Measurement

A JC-1 kit (C2006, Beyotime, Shanghai, China) was used to determined alterations of MMP qualitatively, following the manufacturer’s instructions. Briefly, cells were cultured in 6-well plates, and then incubated with 10 mg/ml MitoProbe JC-1 in the dark for 30 min at 37°C.

### Mitochondrial Morphology Detection in the Cells

To determine mitochondrial fission, NMCMs were cultured on coverslips. The cells were then incubated with MitoTracker Red (M22425, Thermo Fisher Scientific, Waltham, MA, United States) at 37°C for 30 min. The cells were washed with phosphate-buffered saline (PBS) and then were fixed with 4% formaldehyde for 15 min. Nuclei were stained with DAPI. Images were acquired using a confocal laser scanning microscope (LSM710; Carl Zeiss AG, Jena, Germany). Mitochondrial morphology was determined using ImageJ software. Mitochondrial fragmentation vs connectivity was determined by plotting length*width of several thousand mitochondria from >10 cells (Anderson et al., 2018).

### Statistical Analyses

All data are given as the mean ± the standard error of the mean (SEM). Unpaired Student’s t-test and one-way analysis of variance (ANOVA) followed by Turkeys post hoc test were used to analyze the data using GraphPad Prism V.8.0 software (GraphPad Inc., La Jolla, CA, United States). *p* < 0.05 defined statistical significance.

## Results

### Fasudil Improved DM-Induced Cardiac Dysfunction

**FIGURE 1:**
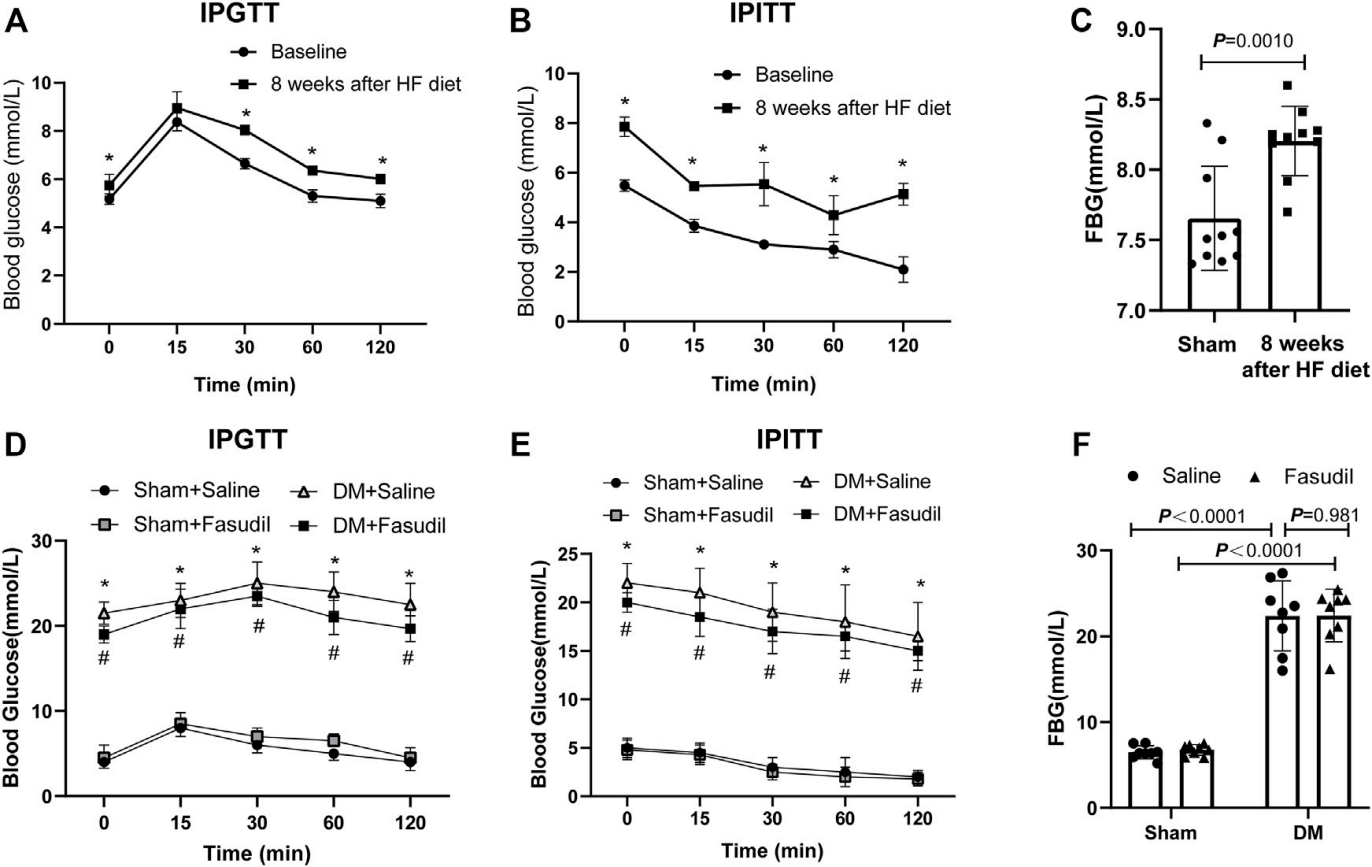
Establishment of type 2 diabetes model of mice **(A–C)** 6 to 8 week old male c57 mice were fed high fat diets for 8 weeks and showed insulin resistance. Intraperitoneal glucose tolerance test (IPGTT), intraperitoneal insulin tolerance test (IPITT), and Fasting blood glucose (FBG) were performed at baseline and at week eight in the DM group (n = 8–10); After 8 weeks of an HF diet, mice with insulin resistance were administered with STZ (i.p.). At the end of experiment, the pathologies of the mice were identified using the following tests **(D)** IPGTT **(E)** IPITT **(F)** FBG. *p*-values were adjusted for comparisons of multiple means. *p < 0.05, **p < 0.01 vs. the Sham + Saline group. ^#^ *p* < 0.05, ^##^ *p* < 0.01 vs. the DM + Saline group.

To achieve the type II diabetes mice model, the mice in diabetic group were given a HF diet to induce insulin resistance. After an 8-weeks HF diet, IPGTT showed that the blood glucose in the diabetic group were higher than normal at all points in time except at 15 min (Figure 1A,C). Similarly, IPITT revealed reduced insulin sensitivity (Figure 1B). these data showed that the diabetic group had insulin resistance after an 8-weeks HF diet. At the end of the experiment, IPGTT, IPITT and FBG in the diabetic group were higher than those in the control (*p* < 0.05) (Figure 1D–F). These findings showed that the diabetic model induced by a HF diet and low-dose STZ had insulin resistance and metabolic disturbance, similar to type II diabetes in humans.

**FIGURE 2:**
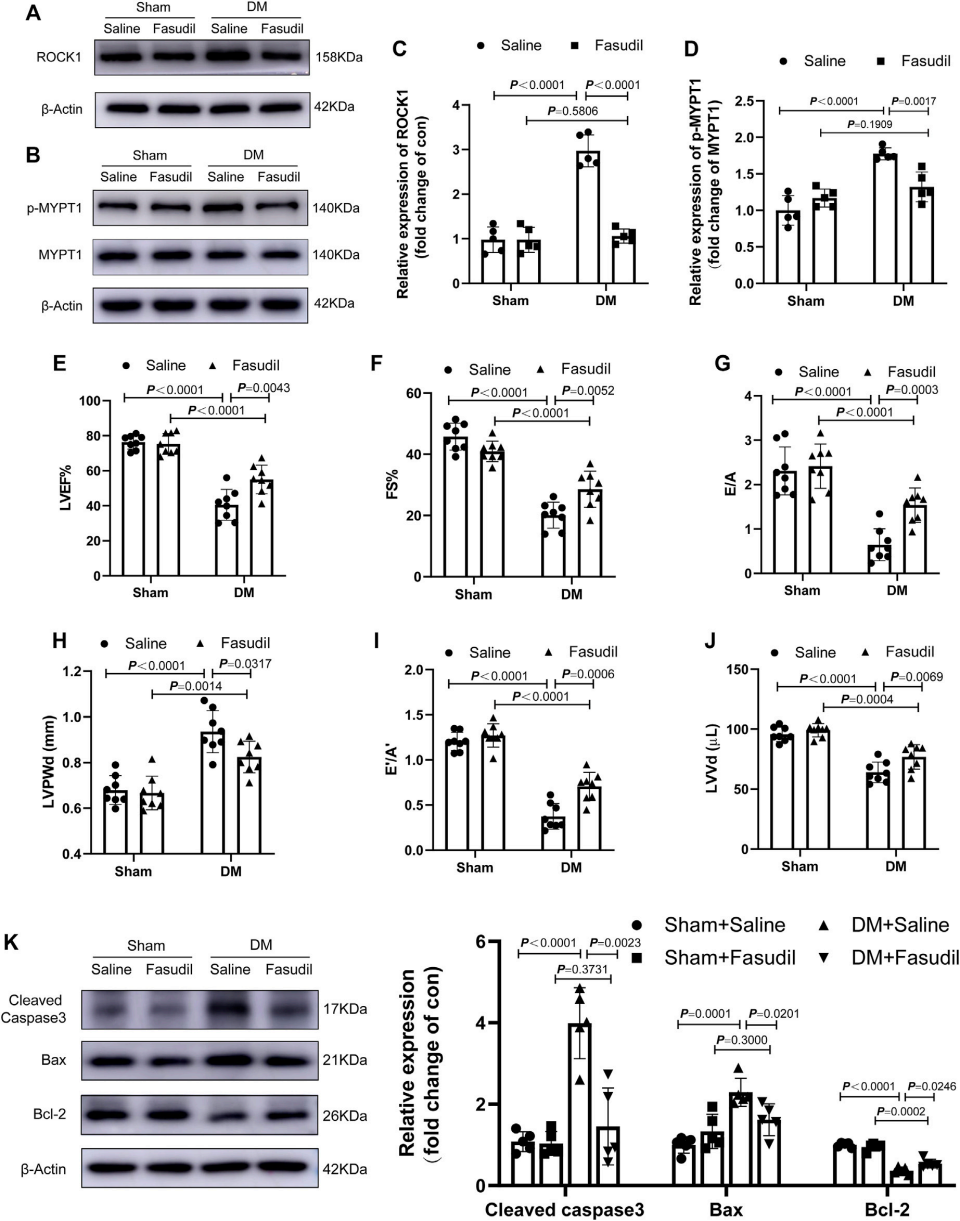
Fasudil improved DM-induced cardiac dysfunction **(A–D)** Representative immunoblots and quantification of ROCK1 and p-MYPT1 in the heart tissues of mice (n = 5) **(E–J)** Left ventricular ejection fraction (LVEF), fractional shortening (FS), and early (E′) to late (A′) diastolic velocity ratio (E’/A′), peak E to peak A ratio (E/A), left ventricle end-diastolic volume (LVVd), as well as left ventricle end-diastolic posterior wall diameter (LVPWd), were evaluated using echocardiography (n = 8) **(K)** Representative immunoblots and quantification of Cleaved Caspase3, Bax and Bcl-2 in the heart tissues of mice (n = 5). *p*-values were adjusted for comparisons of multiple means.

Activated ROCK1 phosphorylates its downstream target MYPT-1, which is one of the best known substrates of ROCK1 (Kawano et al., 1999). In this study, we evaluated the activation (phosphorylation) of ROCK1 by examining MYPT-1 phosphorylation. Fasudil administration restored the increased expression and activation of ROCK1 in diabetic hearts (Figure 2A–D). Concomitantly, the expression of Cleaved Caspase3, Bax and Bcl-2 in the diabetic hearts increased significantly, while Fasudil treatment ameliorated this change (Figure 2K). Compared with that of the sham mice, the cardiac dysfunction of diabetic mice had developed significantly. Echocardiography, including LVEF, E’/A’, FS, E/A ratios, LVPWd, as well as LVVd revealed worse conditions in the DM mice relative to that in the sham group. After treatment, the above-mentioned cardiac performance indexes were improved in the DM + Fasudil group compared with those in the DM + saline group (Figure 2E–J). The above results indicated Fasudil attenuated diabetes induced heart dysfunction and reduced apoptosis in cardiomyocytes.

### Fasudil Reduced Apoptosis, Myocardial Fibrosis, Oxidative Stress and Mitochondrial Damage in Diabetic Mice

**FIGURE 3:**
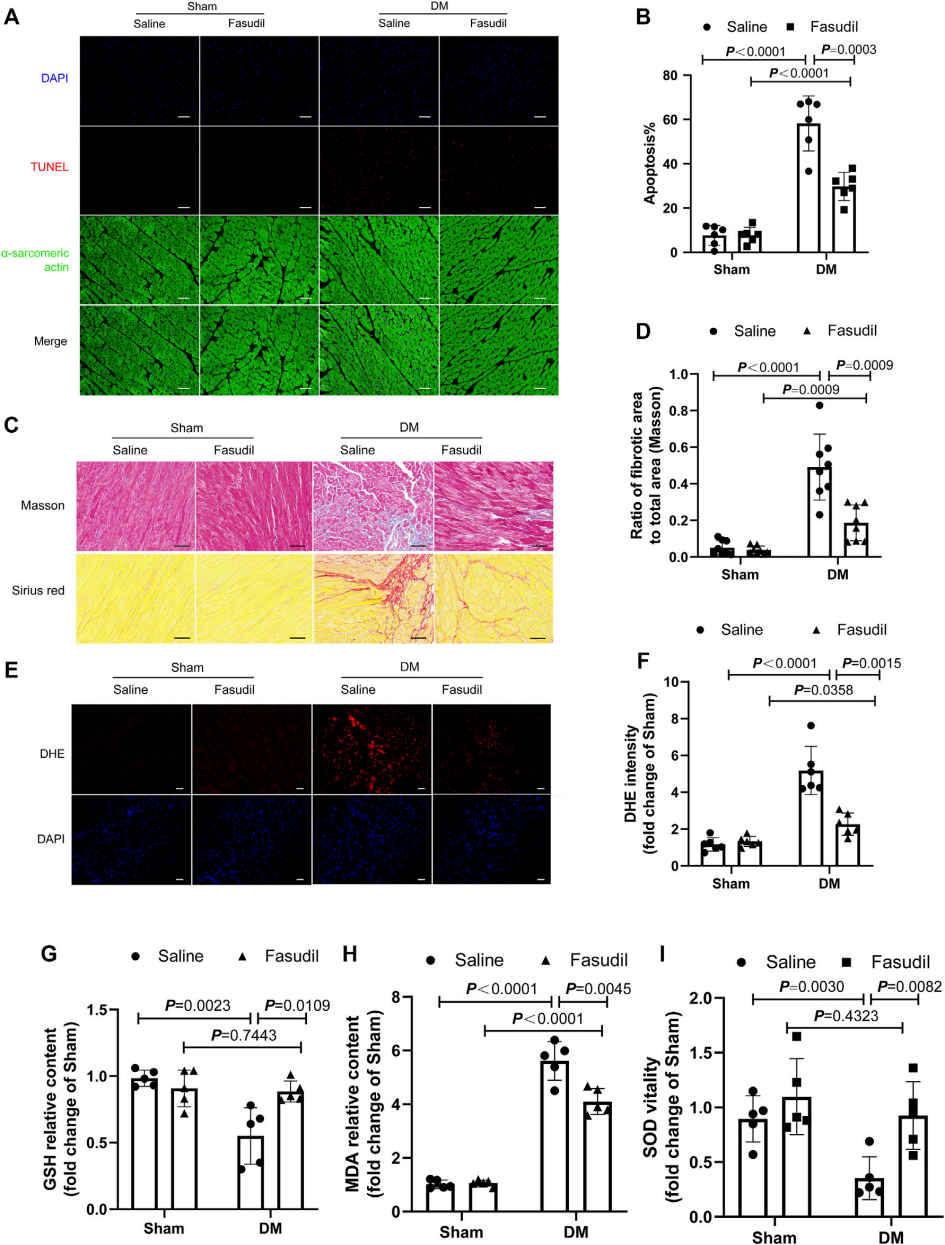
Fasudil improved DM-induced myocardial apoptosis, fibrosis and oxidative stress **(A,B)** Representative sections showing TUNEL and anti-alpha-sarcomeric actin staining and the quantification of TUNEL. Cardiomyocyte apoptosis was analyzed quantitatively (n = 5). Scale bar = 100 μm **(C,D)** Images illustrating Masson and Sirius red staining and quantification. Ratio of the fibrotic area to the total area was analyzed quantitatively. Scale bar = 50 μm (n = 8) **(E,F)** Representative sections of dihydroethidium (DHE) staining and quantification (n = 6). Scale bar = 100 μm **(G–I)** Heart tissue GSH, MDA, and SOD were determined (n = 5). *p*-values were adjusted for comparisons of multiple means.

Diabetic hearts are characterized by apoptosis, myocardial fibrosis and excessive oxidative stress (Bugger et al., 2012). Immunofluorescence staining showed that, in the heart tissue of diabetic mice, the number of apoptotic cell nuclei increased, and Fasudil treatment decreased the apoptosis rate (Figure 3A,B). Similarly, Fasudil treatment decreased the fibrotic areas in the DM mice (Figure 3C,D). As expected, intracellular ROS levels were detected using DHE staining *in vivo* illustrated that the ROS content in the diabetic group was elevated compared with that in the sham mice (*p* < 0.01). The ROS contents in the diabetic mice that were treated with Fasudil decreased markedly compared with those in the untreated diabetic mice (*p* < 0.01, Figure 3E,F). Myocardial GSH, MDA levels, and SOD activities were markedly changed in the diabetic mice versus those in the controls (Figure 3G–I), while they were restored in the Fasudil-treated group. Collectively, the gained by studying type II DM *in vivo* supported the possible participation of ROCK1 in the process of cardiomyocyte apoptosis, fibrosis and oxidative stress.

**FIGURE 4:**
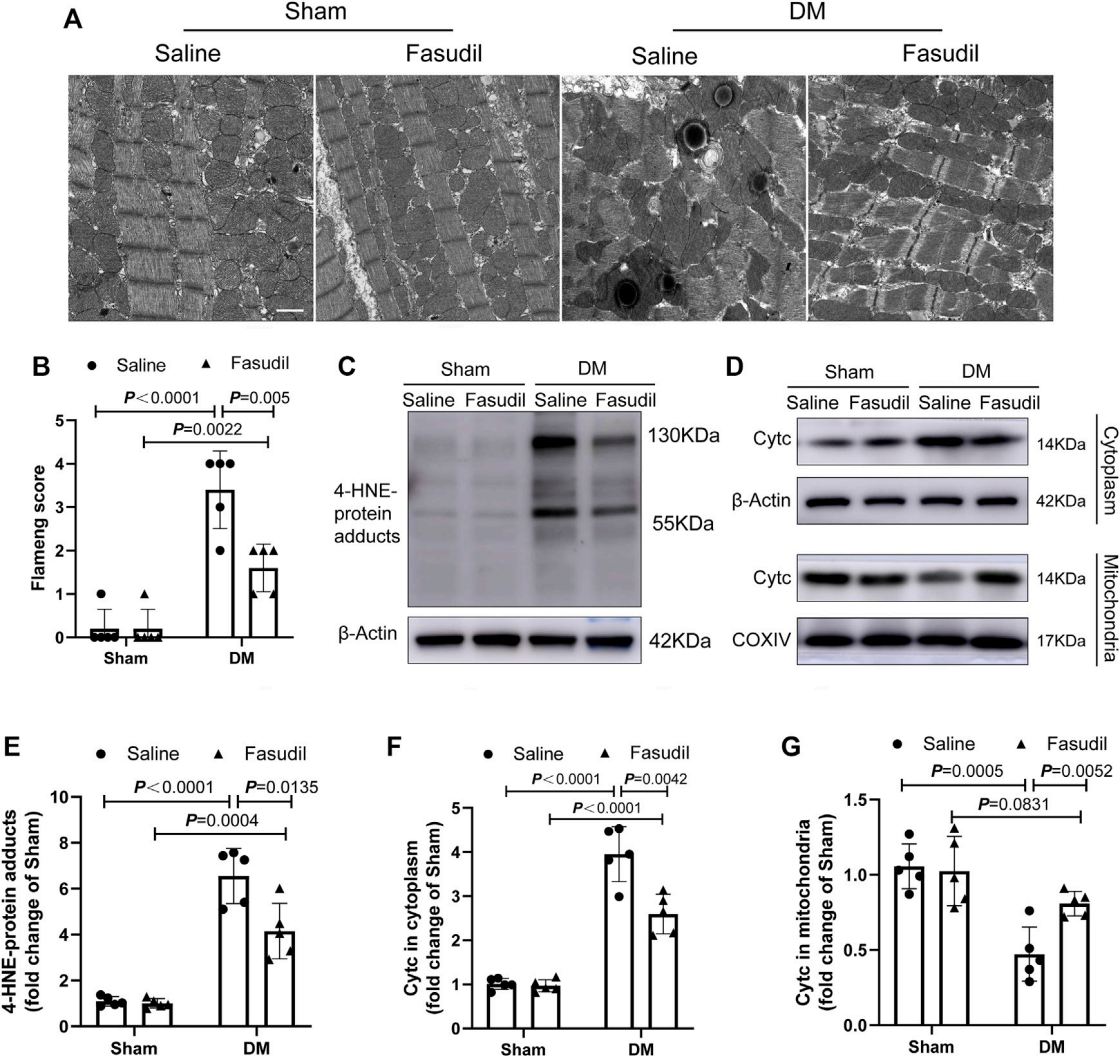
Fasudil reduced mitochondrial dysfunction in the heart of diabetic mice **(A,B)** Representative sections and quantification of mitochondrial morphology by TEM of mouse hearts (n = 5). Scale bar = 1 μm **(C,E)** Immunoblotting and the relative quantitative assessment of 4-HNE-protein adducts levels (n = 5) **(D,F,G)** Representative immunoblots and quantification of cytoplasmic Cytc protein levels and mitochondrial Cytc levels (n = 5). *p*-values were adjusted for comparisons of multiple means.

Mitochondria is a major source of ROS, and substantially involved in oxidative stress. Thus we evaluated myocardial ultrastructure and mitochondrial structure by electron microscopy (TEM). TEM analysis revealed well-organized myofibrils and regular Z-lines in the sham group. By contrast, the ultrastructure of the left ventricular of the DM mice was severely damaged, including disruption of myofibrils, abnormal Z-lines, swelling of mitochondria with disordered arrangement, and lipid accumulation. Fasudil treatment improved the cardiac ultrastructure and mitochondria morphology in the DM mice (Figure 4A,B). The elevated four HNE-protein adducts (mitochondrial damage markers) and cytochrome c (the crucial regulator of the mitochondrial apoptotic pathway) in the cytoplasm of cells from the DM mice were also inhibited by Fasudil (*p* < 0.01, Figure 4C–G). These results suggest that the improvement of mitochondrial dysfunction might be the important regulatory mechanism by which Fasudil alleviated oxidative stress injury in DCM.

### Fasudil Mitigated Cardiomyocyte Apoptosis Induced by HG *in vitro*

**FIGURE 5:**
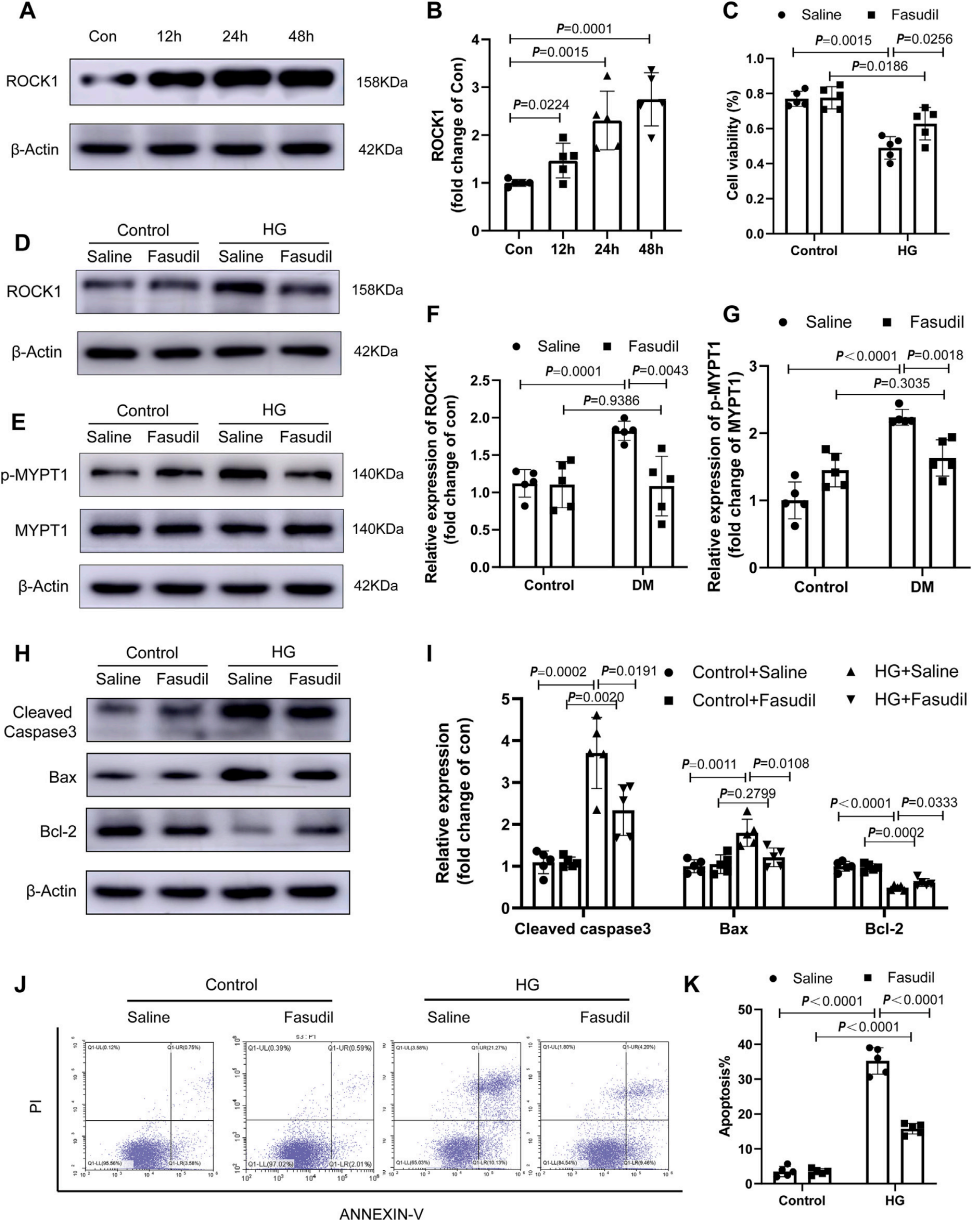
Fasudil ameliorated cardiomyocyte apoptosis induced by hyperglycemia and hyperinsulinemia *in vitro* **(A,B)** Immunoblotting and the relative quantitative assessment of ROCK1 levels in NMCMs treated high glucose (33 mM) and insulin (10 nmol/L) for different times (12–48 h) (n = 5) **(C)** The effect of Fasudil in response to HG on the proliferation index of cardiomyocytes using the CCK8 assay (n = 5) **(D–G)** Immunoblotting and the relative quantitative assessment of ROCK1 and p-MYPT1 in NMCMs treated with HG or HG + Fasudil (100 μM) treatment for 48 h (n = 5) **(H,I)** Representative immunoblots and quantification of Cleaved Caspase3, Bax and Bcl-2 in NMCMs treated with HG or HG + Fasudil (100 μM) treatment for 48 h (n = 5) **(J,K)** Apoptosis detection and quantitative analysis via Annexin V/PI staining of cardiomyocytes in HG conditions or Fasudil treatment for 48 h (n = 5). *p*-values were adjusted for comparisons of multiple means.

NMCMs were observed by fluorescence microscopy using cardiac troponin I (cTnI) as a specific cardiomyocyte protein (Supplementary Figure S1). We examined an insulin concentration gradient (Supplementary Figure S2) and time point experiments (Figure 5A). *In vitro*, ROCK1 expression in NMCMs increased remarkably at 24 h (*p* < 0.05) and 48 h (*p* < 0.01) in the HG group (Figure 5A,B), and the activation of ROCK1 increased under 48 h-HG treatment (Figure 5E,G). To test whether ROCK signaling is important for cardiomyocyte apoptosis, we treated NMCMs with the Fasudil. The protein levels and activation of ROCK1 were lower in HG-treated NMCMs under Fasudil treatment compared with those in the control (Figure 5D–G). CCK8 assay showed that Fasudil restored the proliferative ability of cardiomyocytes impaired by HG treatment (Figure 5C). The expression of apoptosis-related proteins, including cleaved caspase3, Bax and Bcl-2, and flow cytometric analysis showed that HG-induced cardiomyocyte apoptosis was increased significantly *in vitro*, while Fasudil markedly inhibited apoptosis in cardiomyocytes (Figure 5H–K). Altogether, these results supported the view that Fasudil alleviated HG-induced apoptosis of cardiomyocytes.

### Fasudil Abrogated Mitochondrial Fission and Superoxide Generation in Hyperglycemic Cardiomyocytes

**FIGURE 6:**
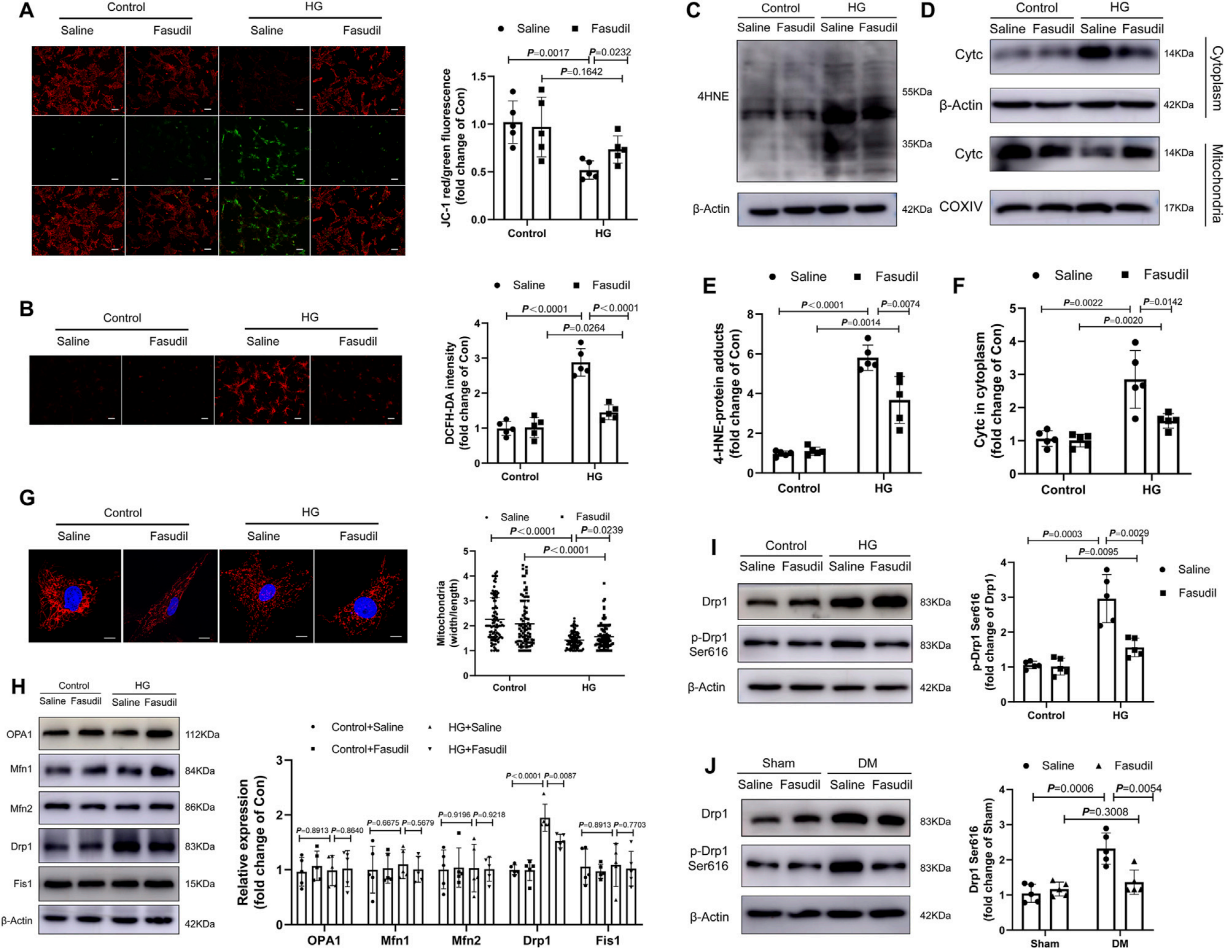
Fasudil abrogated mitochondrial fission and superoxide generation in hyperglycemia-treated cardiomyocytes **(A)** The MMP was measured using JC-1 staining. The histograms indicate the ratio of green and red fluorescence (n = 5) **(B)** DCFH-DA staining and quantitative evaluation of ROS in NMCMs with HG treatment and Fasudil (10 μM) treatment for 48 h (n = 5) **(C,E)** Immunoblotting and quantitative assessment of 4-HNE in NMCMs (n = 5) **(D,F)** Cytoplasmic Cytc protein levels, mitochondrial Cytc protein levels in NMCMs under 48 h periods of HG stimulation and Fasudil treatment (n = 5) **(G)** NMCMs were treated with HG (33 mM and 10 nmol/L insulin) and stained using Mitotracker Red and DAPI (n = 3). Confocal laser scanning fluorescence microscope imaging of the mitochondrial morphology. Mitochondrial fission was evaluated by the ratio of mitochondrial length and width, which was plotted for thousands of mitochondria from >10 cells. Scale bar = 20 μm **(H)** Immunoblotting and quantitative assessment of OPA1, Mfn1, Mfn2, Drp1, Fis1 in NMCMs under 48 h periods of HG stimulation and Fasudil treatment (n = 5) **(I)** levels of Drp1 phosphorylated at serine 616 in NMCMs under 48 h periods of HG stimulation and Fasudil treatment (n = 5) **(J)** Representative immunoblots and quantification of the levels of Drp1 phosphorylated at serine 616 *in vivo* (n = 5). *p*-values were adjusted for comparisons of multiple means.

As we have known, MMP is related to the mitochondrial apoptotic pathway. The changes to the MMP were analyzed using fluorescence microscopy with JC-1 staining. As shown in Figure 6A, MMP was reduced significantly in cardiomyocytes that were treated with HG for 48 h (*p <* 0.01); however, the MMP almost returned to normal levels after Fasudil treatment (*p <* 0.05). Similarly, the cellular ROS levels were elevated during HG treatment, but were reduced by Fasudil (Figure 6B). The levels of 4-HNE-protein adducts and cytochrome c in the cytoplasm of cardiomyocytes were increased after HG-treatment, but were decreased by Fasudil treatment (Figure 6C–F). These results reconfirmed the beneficial effect of Fasudil on improving HG-induced mitochondrial dysfunction *in vitro*.

Mitochondrial dynamics are closely connected with mitochondrial dysfunction, as well as activation of oxidative stress triggered by HG (Ding et al., 2018a; Shi et al., 2018). Therefore, we analyzed the morphology of mitochondria using MitoTracker (Red) staining *in vitro*, and investigated whether Fasudil reduces mitochondrial fragmentation in NMCMs under elevated glucose conditions. As shown in Figure 6G, after induction with elevated glucose for 48 h, the mitochondria had shrunk, and their number had increased, indicating that the mitochondrial network was fragmented. ROCK1 inhibitor Fasudil prevented HG-triggered mitochondrial fragmentation.

Meanwhile, we analyzed protein expressions related to mitochondrial fission and fusion-associated under HG condition. However, there were no changes in OPA1 (optic atrophy protein 1), MFN (mitofusin)1, MFN2 and Fis1 (mitochondrial fission protein 1) proteins expression levels in HG group compared with those in the control group (Figure 6H). Compared with the control group, HG induced Drp1 (dynamin-related protein 1) and activated Drp1 (Ser616-phosphorylated Drp1) increased significantly (Figure 6I). Notably, Fasudil treatment reversed HG-induced mitochondrial fragmentation (Figure 6G) and reduced phosphorylation of Drp1 at Ser616 (Figure 6J). This *in vitro* result was confirmed in the mouse model of type 2 diabetes (Figure 6J). These data showed that hyperglycemia increased Drp1 phosphorylation at Ser616 mediated mitochondrial fission, while Fasudil treatment decreased the level of p-Drp1 Ser616 enhanced by diabetes.

**FIGURE 7:**
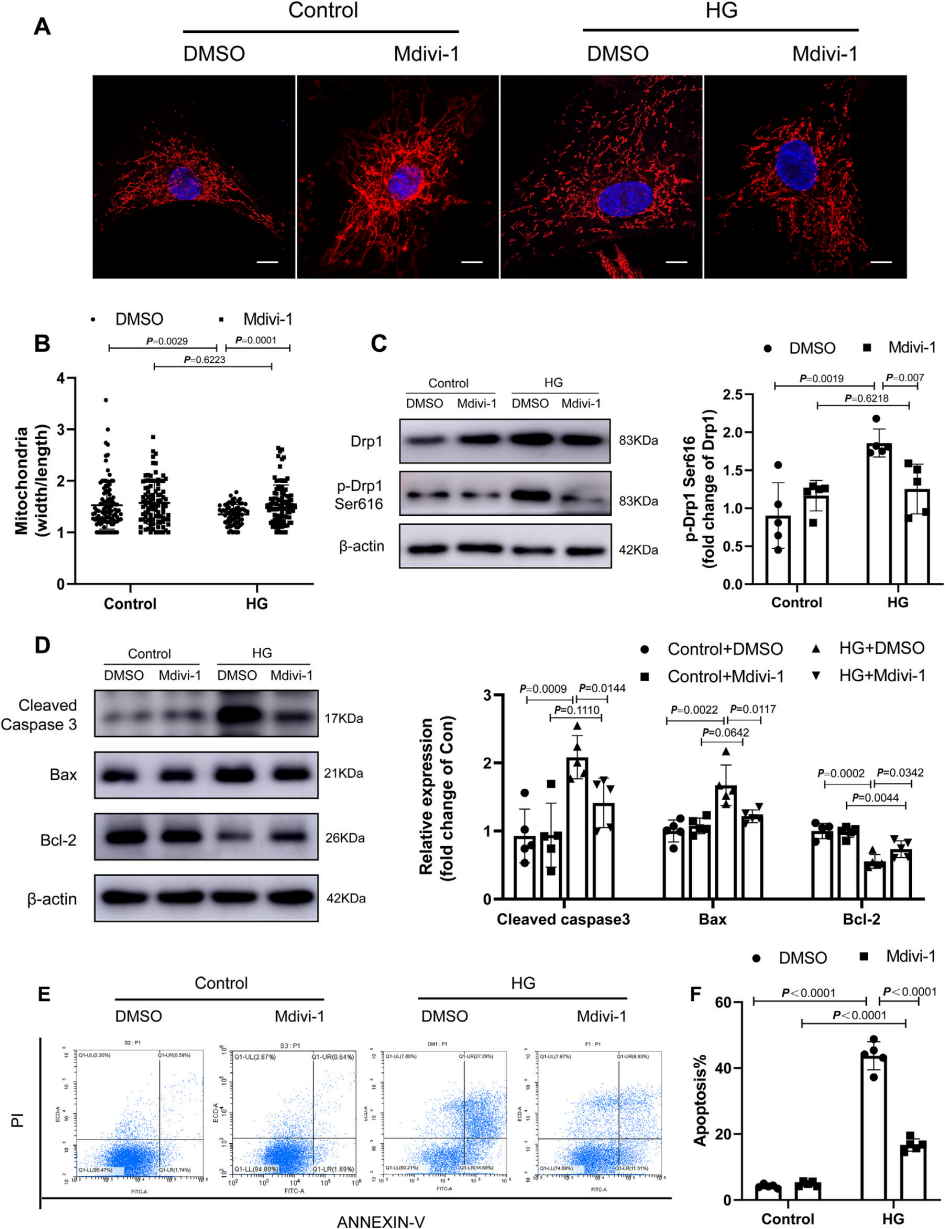
Fasudil ameliorated cardiomyocyte apoptosis induced by hyperglycemia and hyperinsulinemia *in vitro*. **(A,B)** NMCMs were treated with HG (33 mM and 10 nmol/L insulin) and stained using Mitotracker Red and DAPI (n = 3). Confocal laser scanning fluorescence microscope imaging of the mitochondrial morphology. Mitochondrial fission was evaluated by the ratio of mitochondrial length and width, which was plotted for thousands of mitochondria from >10 cells. Scale bar = 20 μm **(C)** levels of Drp1 phosphorylated at serine 616 in NMCMs under 48 h periods of HG stimulation and Mdivi-1 treatment (n = 5) **(D)** Representative immunoblots and quantification of Cleaved Caspase3, Bax and Bcl-2 in NMCMs under 48 h periods of HG stimulation and Mdivi-1 treatment (*n* = 5) **(E,F)** Apoptosis detection and quantitative analysis via Annexin V/PI staining of cardiomyocytes in HG conditions or Mdivi-1 treatment for 48 h (n = 5). *p*-values were adjusted for comparisons of multiple means.

To confirm the role of Drp1 Ser616 phosphorylation in HG-induced cardiomyocyte apoptosis, the selective inhibitor of Drp1, Mdivi-1 was used to block Drp1 activity (Figure 7C). Mitochondrial fission was suppressed by Drp1 inhibition in HG condition (Figure 7A,B). Importantly, Drp1 inhibition prevented the effect of HG on the proliferation and cardiomyocyte apoptosis (Figure 7D–F).

**FIGURE 8:**
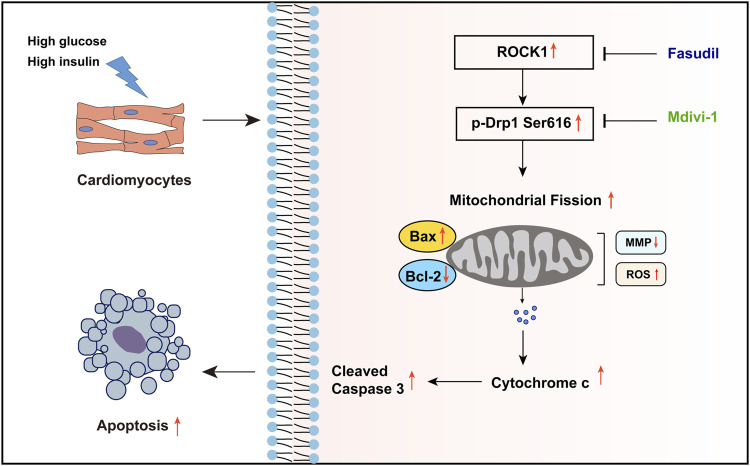
Summary of the mechanisms of Fasudil that inhibit excessive mitochondrial fission in DCM. The high glucose and high insulin environment caused by type 2 diabetes stimulates the expression and activity of ROCK1 in cardiomyocytes, and increases the phosphorylation of its downstream Drp Ser616 site, which leads to excessive mitochondrial fission and ultimately increases cardiomyocyte apoptosis. Fasudil can inhibit the expression and activity of ROCK1, thereby reducing cardiomyocyte apoptosis.

Taken together, the increased expression and activity of ROCK1 during DCM leads to increased phosphorylation of Drp1 on Ser616, which promoted mitochondrial fission. Excessive mitochondrial fission generated oxidative stress and resulted in mitochondrial dysfunction. The decrease in MMP resulted in the release of the pro-apoptotic factor Cytc from the mitochondrial matrix into the cytoplasm, activated the caspase-dependent apoptotic pathways, and ultimately leading to cardiac dysfunction in DCM. Fasudil reverses this process by inhibiting the expression and activity of ROCK1, thereby exerting a protective effect against type 2 diabetes-induced cardiac dysfunction (Figure 8).

## Discussion

The pathogenesis of DCM remains unclear, and there is lack of effective treatment. In the present study, we aimed to explore the effects of a ROCK1 inhibitor, Fasudil in DCM and to determine its underlying mechanisms. We found that the content of ROCK1 protein in the diabetic mice heart tissue was elevated. Then we determined whether Fasudil played a protective effect against type II diabetes-induced cardiomyocyte apoptosis *in vivo*, as well as *in vitro*. We next investigated the downstream effectors of Fasudil that might impact DCM. Our *in vitro* and *in vivo* data illustrated that ROCK1 induces mitochondrial fission in diabetic mice by activating Drp1 by phosphorylation at Ser616, and inhibition of ROCK1 with Fasudil reversed the above effects and played a role in cardioprotective effects in DCM.

Most patients with diabetes suffer from T2DM, which is characterized by insulin resistance and hyperglycemia. Hyperglycemia enhances insulin resistance, which in turn, leads to adverse cardiac events (Bugger et al., 2012). Similar to previous research, our DM mice showed DM cardiomyopathy, which is manifested by systolic and diastolic dysfunction, the alteration of myocardial ultrastructure, and fibrosis. Treatment with Fasudil was effective to treat the cardiac dysfunction. The protective influence provided by Fasudil depended on reducing the pathological changes of myofibrils, mitochondria fission, and cardiomyocyte apoptosis.

ROCK1 belongs to the serine/threonine protein kinase family, and is a key effector of the small GTPase, RhoA (Shimokawa et al., 2016). It has an indispensable role in regulating cell polarity, morphology, cytoskeleton remodeling, migration and adhesion by regulating actin (Nunes et al., 2010). Research has confirmed that ROCK1 plays a vital role in cardiovascular disease. HG could increase ROCK1 expression and activate RhoA/ROCK1 (Massey et al., 2003; Peng et al., 2008; Hirose et al., 2010). The activation of ROCK1 has been demonstrated to cause diabetic systemic inflammation and abnormal protein synthesis (Lu et al., 2014). Here, we provided evidence that HG could upregulate the ROCK1 protein level in cardiomyocytes, and treatment with Fasudil significantly reduced the expression and activation of ROCK1 induced by type II diabetes. However, in addition to acting as a ROCK1 inhibitor, Fasudil also could interfere with the function of ROCK2 (another isoform of ROCK family), which is preferentially expressed in the brain (Nakagawa et al., 1996). We will further focus on the effect of ROCK2 inhibition on type 2 diabetes mice in a follow-up study.

Mitochondria are pivotal organelles in cells, forming the core of energy metabolism, and the center of cell modulation of oxidative stress. Increasing evidence shows that mitochondrial dysfunction participates in the pathogenesis of cardiac dysfunction caused by diabetes (Mariappan et al., 2010; Verma et al., 2017). Mitochondrial dysfunction is related strongly to the upstream pathways in various pathogenic mechanisms, involving energy generation, ROS production, and cell death processes (Willems et al., 2015; Nan et al., 2017; Verma et al., 2017; Ding et al., 2018b). Meanwhile, mitochondria are the major location of ROS production and the main target of ROS. Under pathological conditions, cell energy metabolism is disordered, and the generation of excessive ROS can result in oxidative damage to mitochondria by oxidizing cardiolipin, mtDNA, and important mitochondrial proteins. Oxidative stress can decrease the MMP, increasing the permeability of transition pores in the mitochondrial membrane, and changing mitochondrial membrane permeability, which causes the release Cytc and other pro-apoptotic factors (Zhang et al., 2015). Release of Cytc activates the apoptotic pathway of the caspase cascade, resulting in cell apoptosis.

Elevated ROS generation is recognized as the main mechanism of heart failure in individuals with diabetes. In the case of hyperglycemia, mitochondrial fission is recognized as a key source of ROS (Brooks et al., 2009; Bravo-San Pedro et al., 2017). As highly dynamic organelles in the cell, mitochondria regulate their number, shape, and distribution through continuous division and fusion, which determine indirectly the function and even survival of cells. This process is regulated finely by a variety of proteins (Westermann, 2010; Whitley et al., 2019). Under pathological conditions, the mitochondrial fission and fusion dynamic balance is disrupted and further leads to mitochondrial dysfunction. Drp1 is an important cytoplasmic GTPase, and its phosphorylation can promote significant mitochondrial division. A previous investigation showed that HG stimulation results in an increased level of Drp1 in many types of cells, including cardiomyocytes (Brooks et al., 2009). However, whether mitochondrial fission participates in the pathogenesis of T2DM-triggered cardiac dysfunction *in vivo* is unknown. Our research showed that ROCK1 could modulate the mitochondrial dynamics of cardiomyocytes, and overexpression of ROCK1 increased mitochondrial fission. In diabetic nephropathy, Drp1 was identified as a direct substrate for ROCK1, and ROCK1 was proved to mediate phosphorylation of Drp1 at Ser600 (Wang et al., 2012). However, our findings do not exclude the possibility that other ROCK1-dependent pathways, independent regulation of Drp1 phosphorylation, are also involved in the ROCK1-mediated cellular adaption to metabolic stress. And whether ROCK1 directly phosphorylates Drp1 or indirectly dephosphorylates Drp1 through other phosphatases may be great variability between different cell lines (Zhang et al., 2019). In general, we introduce a novel concept that mitochondrial fission caused by hyperglycemia participates in cardiac dysfunction in mice with type 2 diabetes, partially by enhancing oxidative stress and impairing mitochondrial function, thus opening mitochondrial membrane pores. This leads to a decreased MMP and the release of apoptosis-related proteins, such as Cytc, eventually leading to cardiomyocyte apoptosis.

There are some limitations within our study. First, we studied the effect of Fasudil on cardiomyocyte apoptosis under hyperglycemia and hyperinsulinemia conditions *in vitro*. However, the impact of Fasudil on cardiomyocytes in hyperlipidemia condition was not assessed, which is the goal for our further study. Second, the further underlying mechanisms between Drp1 and ROCK1 on mitochondrial fission in cardiomyocytes represents a major area for our future research. Third, we used NMCMs rather than primary adult mice cardiomyocytes to investigate the effects of Fasudil *in vitro*. Because the adherent time of adult mice cardiomyocytes is less than 12 h in our lab due to technical reasons, it is not possible to carry out our experiments in adult mice cardiomyocytes.

In summary, our data defined a ROCK1-dependent cascade through which ROCK1 enhances mitochondrial fragmentation in elevated glucose conditions, and identified cardiac mitochondrial dynamics regulated by Fasudil as a prospective treatment approach for cardiac dysfunction, as well as other cardiac complications in patients with diabetes.

## Data Availability

The raw data supporting the conclusions of this article will be made available by the authors, without undue reservation.
