# Supplementary material for: A ROCK1 Inhibitior Fasudil Alleviates Cardiomyocyte Apoptosis in Diabetic Cardiomyopathy by Inhibiting Mitochondrial Fission in a Type 2 Diabetes Mouse Model
Source: Front Pharmacol. 2022 Jul 5;13:892643. doi: 10.3389/fphar.2022.892643 (PMC9294374; doi:10.3389/fphar.2022.892643)
Supplement: Supplementary file 1 [file DataSheet1.docx]

Supplementary Material

## Supplementary Figures


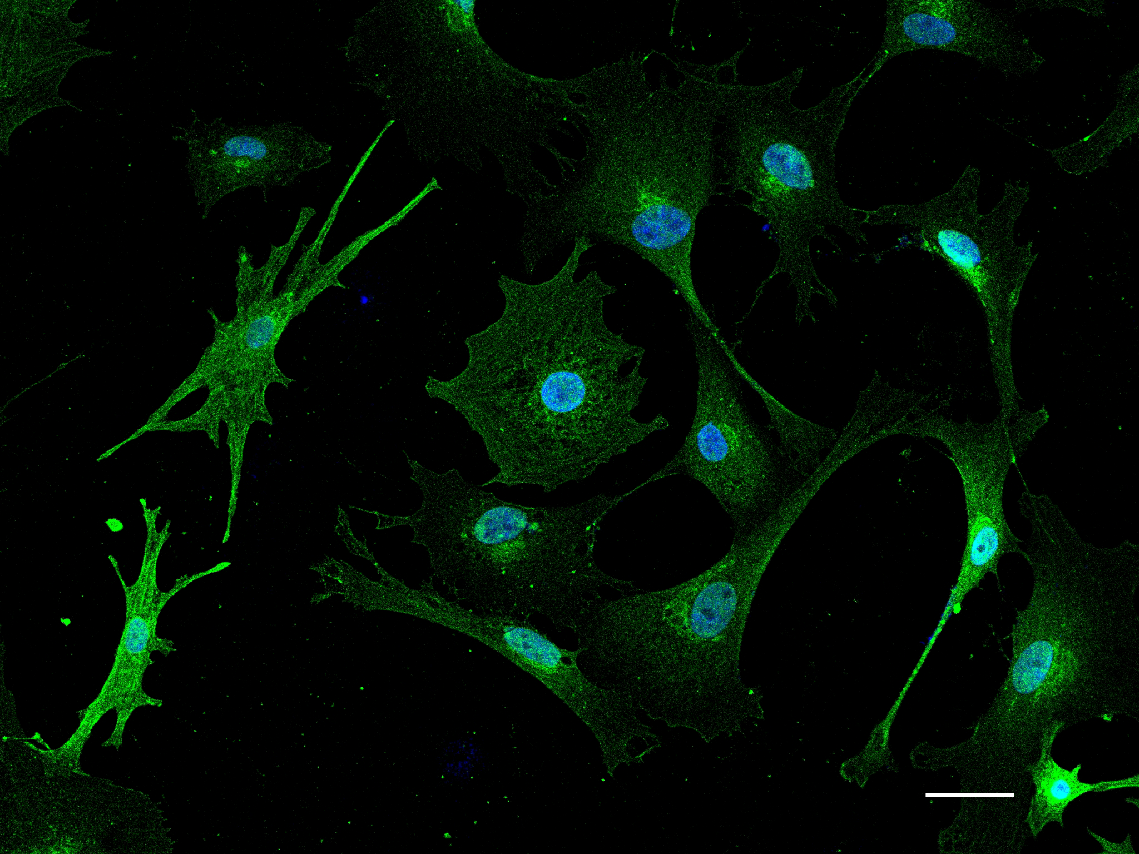


**Supplementary Figure 1.** **Immunofluorescence identification of primary cardiomyocytes**

1. Neonatal mouse ventricular cardiac myocytes (NMCMs) were stained using cardiac troponin I (cTnI) and DAPI. CTnI is the marker protein for cardiac myocytes. Blue fluorescence represents DAPI staining and green fluorescence represents cTnI. Scale bar = 20 μm.

**
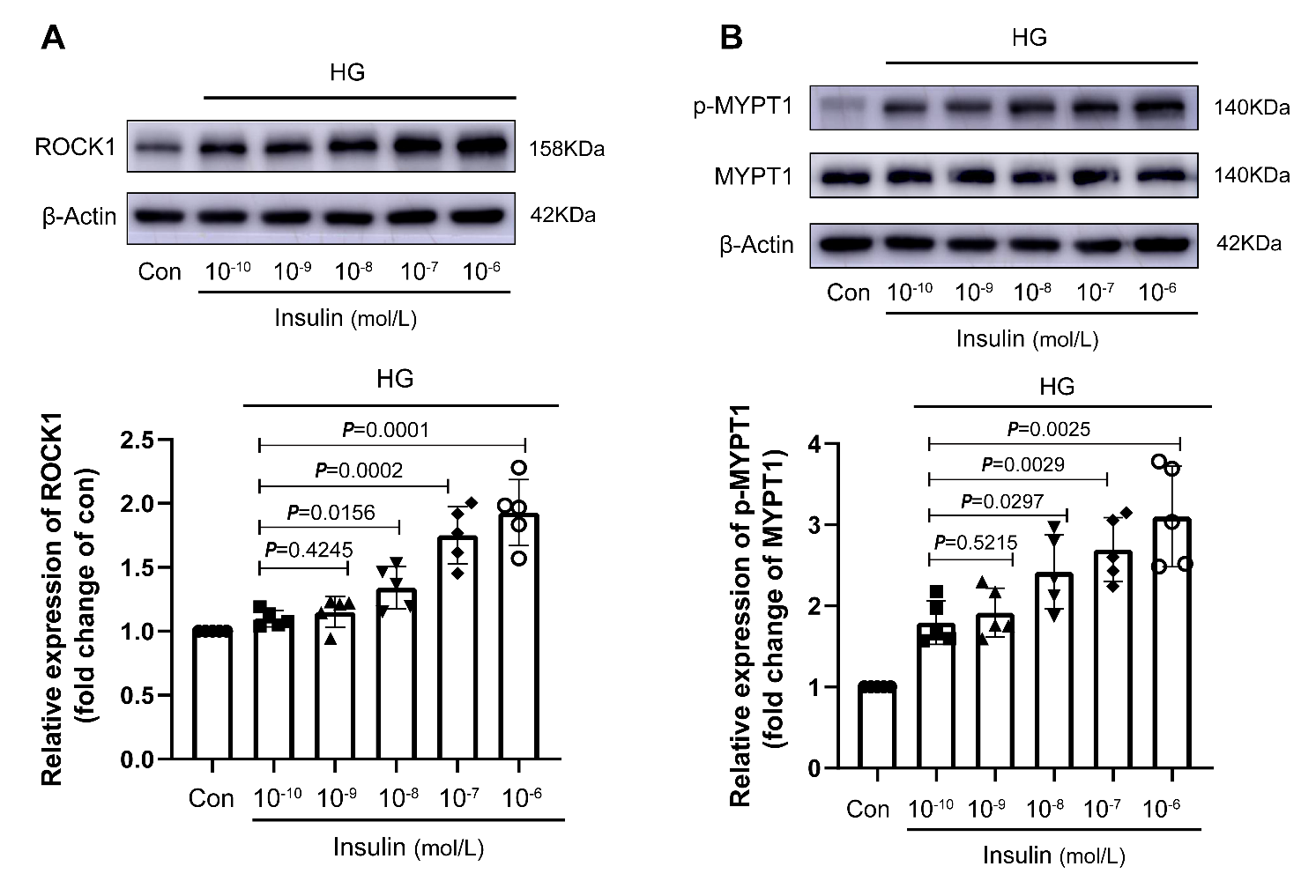
**

**Supplementary Figure 2.** **The insulin concentration gradient experiments in neonatal mouse ventricular cardiac myocytes (NMCMs).**

Cardiomyocytes were treated with 33 mM in DMEM in the absence or presence of various doses of insulin. (A) Effects of high glucose and insulin on ROCK1 expression in NMCMs (n = 5). (B) Effects of high glucose and insulin on ROCK1 activity in NMCMs (n = 5).
